# High throughput screening identifies modulators of histone deacetylase inhibitors

**DOI:** 10.1186/1471-2164-15-528

**Published:** 2014-06-26

**Authors:** Ann-Christin Gaupel, Thomas Begley, Martin Tenniswood

**Affiliations:** Department of Biomedical Sciences, School of Public Health, University at Albany, New York, 12222 USA; Cancer Research Center, University at Albany, 1 Discovery Drive, Rensselaer, NY 12144 USA; Nanobioscience Constellation, SUNY-College of Nanoscale Science and Engineering, Albany, NY 12203 USA

**Keywords:** Histone deacetylase inhibitor, CG-1521, Yeast deletion library screen, Histone acetyltransferase, SAGA complex, Gcn5, Chromatin remodeling, Transcription

## Abstract

**Background:**

Previous studies from our laboratory and others have demonstrated that in addition to altering chromatin acetylation and conformation, histone deacetylase inhibitors (HDACi) disrupt the acetylation status of numerous transcription factors and other proteins. A whole genome yeast deletion library screen was used to identify components of the transcriptional apparatus that modulate the sensitivity to the hydroxamic acid-based HDACi, CG-1521.

**Results:**

Screening 4852 haploid *Saccharomyces cerevisiae* deletion strains for sensitivity to CG-1521 identifies 407 sensitive and 80 resistant strains. Gene ontology (GO) enrichment analysis shows that strains sensitive to CG-1521 are highly enriched in processes regulating chromatin remodeling and transcription as well as other ontologies, including vacuolar acidification and vesicle-mediated transport. CG-1521-resistant strains include those deficient in the regulation of transcription and tRNA modification. Components of the SAGA histone acetyltransferase (HAT) complex are overrepresented in the sensitive strains, including the catalytic subunit, Gcn5. Cell cycle analysis indicates that both the wild-type and *gcn5Δ* strains show a G_1_ delay after CG-1521 treatment, however the *gcn5Δ* strain displays increased sensitivity to CG-1521-induced cell death compared to the wild-type strain. To test whether the enzymatic activity of Gcn5 is necessary in the response to CG-1521, growth assays with a yeast strain expressing a catalytically inactive variant of the Gcn5 protein were performed and the results show that this strain is less sensitive to CG-1521 than the *gcn5Δ* strain.

**Conclusion:**

Genome-wide deletion mutant screening identifies biological processes that affect the sensitivity to the HDAC inhibitor CG-1521, including transcription and chromatin remodeling. This study illuminates the pathways involved in the response to CG-1521 in yeast and provides incentives to understand the mechanisms of HDAC inhibitors in cancer cells. The data presented here demonstrate that components of the SAGA complex are involved in mediating the response to CG-1521. Additional experiments suggest that functions other than the acetyltransferase activity of Gcn5 may be sufficient to attenuate the effects of CG-1521 on cell growth.

**Electronic supplementary material:**

The online version of this article (doi:10.1186/1471-2164-15-528) contains supplementary material, which is available to authorized users.

## Background

Many human cancers display abnormal post-translational modifications of histones, including acetylation [1–4], and histone deacetylases (HDACs) are known to be aberrantly expressed in a variety of cancer cells [5]. It has been suggested that changes in histone modifications and histone deacetylase expression levels may be useful prognostic indicators of survival and recurrence in a variety of cancers [2–4, 6]. Mammalian HDACs can be subdivided into two families: the classical HDAC family and the sirtuins. The classical HDACs are Zn^2+^ dependent enzymes: class I HDACs (HDACs 1, 2, 3, and 8) share homology to the yeast HDAC Rpd3 and are localized to the nucleus; class II HDACs are related to yeast Hda1 and shuttle between the cytosol and nucleus (HDAC 4, 5, 7, 9) or reside in the cytosol (HDAC 6, 10). HDAC 11 (class IV), homologous to Hos3, resides in the cytosol and nucleus. Over the last 10–15 years a variety of natural and synthetic HDAC inhibitors have been developed, including hydroxamic acid derivatives, benzamides, short chain fatty acids, cyclic tetrapeptides, and electrophilic ketones. Hydroxamic acid derivatives, including Trichostatin A (TSA), suberoylanilide hydroxamic acid (SAHA) and CG-1521 (7-phenyl-2,4,6-hepta-trienoic hydroxamic acid), inhibit the classical family of HDACs by coordinating the catalytic site Zn^2+^, stabilizing the acetylation of histones and non-histone proteins. This induces a variety of responses including cell cycle arrest, cell death, differentiation and senescence, depending on the cell lines and inhibitors used [7, 8]. HDACis are attractive agents because therapeutically active concentrations are minimally toxic to the host and transformed cells are more sensitive to HDACi-induced cell death than normal cells [9–12]. To date, two HDACis, SAHA and Romidepsin, a cyclic tetrapeptide, have been approved by the FDA for the treatment of cutaneous T-cell lymphoma [13, 14]. Previous studies from our laboratory and others have demonstrated that hydroxamic acid-based HDACis have profound impacts on the biology of prostate and breast cancer cell lines, inducing growth arrest and apoptosis [15–19].

The aim of the research reported here was to identify transcription factors that may be useful for novel therapeutic approaches in combination with HDAC inhibitors for hard-to-treat-cancers. We have taken a systems biology approach, screening a *Saccharomyces cerevisiae* haploid single gene deletion library, to identify gene products that modulate the response to HDAC inhibition. *S. cerevisiae* is a valuable model organism for which there is a wide array of information available (including transcriptional profiling, interaction studies and synthetic genetic analysis) to use in analyzing new high throughput data sets [20–23]. Furthermore, histones and histone modifying enzymes show a high degree of sequence and functional conservation among eukaryotes [24–27].

## Results

### CG-1521-sensitive and-resistant strains are enriched for genes involved in chromatin remodeling and transcription

**Figure 1:**
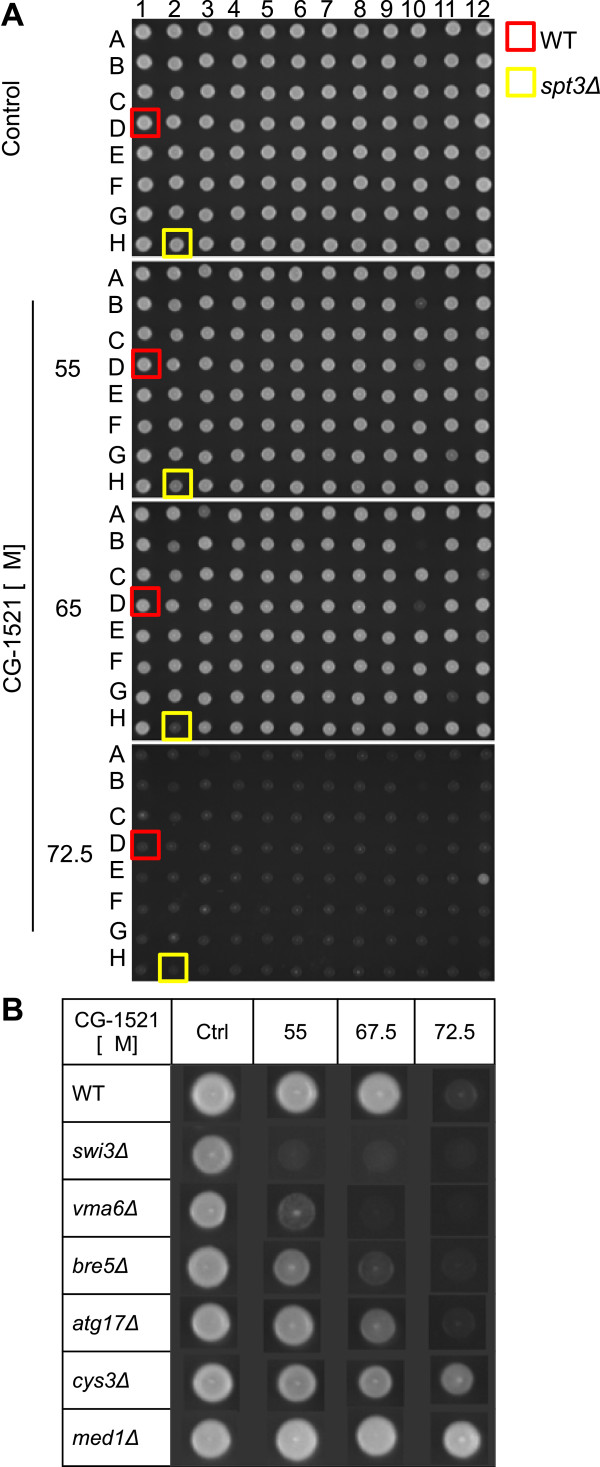
**Genomic phenotyping with CG-1521.** Panel **A**. Representative YPD agar plates. 4852 yeast gene deletion strains, arrayed on 96 well plates, were tested for sensitivity and resistance to CG-1521. Strains, grown to stationary phase, were spotted on agar plates containing the indicated concentrations of CG-1521. Plates were imaged after 60 h. The BY4741 wild-type strain (red) and the CG-1521-sensitive strain *spt3*Δ (yellow) were spotted on each plate as controls. Panel **B**. Examples of strains displaying sensitivity or resistance to CG-1521. Images of wild-type, *swi3*Δ, *vma6*Δ, *bre5*Δ, *atg17*Δ, *cys3*Δ and *med1*Δ were compiled to show varying degrees of sensitivity or resistance to CG-1521.

Genomic phenotyping was performed to detect CG-1521-sensitive and –resistant strains. Gene deletion strains were spotted on agar plates containing low (55 μM), medium (67.5 μM) or high (72.5 μM) concentrations of CG-1521. Strain growth was imaged and sensitive and resistant strains were visually identified. Examples of strains with different grades of sensitivity and resistance are shown in Figure 1. 407 sensitive and 80 resistant gene deletion mutants were identified (Additional file 1). *S. cerevisiae* is more resistant to the hydroxamic acid based HDACi TSA and SAHA. Sensitive strains can only be identified with concentrations starting at 150 μM TSA, while SAHA does not induce changes in growth up to concentrations of 1.75 mM SAHA (data not shown). Due to these limitations, it is not feasible to identify sensitive and resistant strains in response to TSA and SAHA.

**Figure 2:**
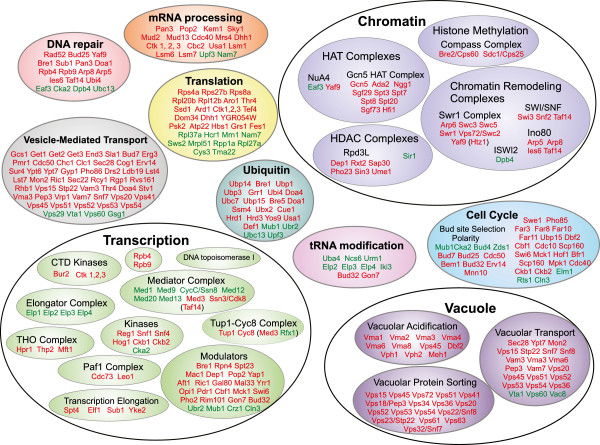
**Functional categorization of CG-1521-sensitive and-resistant strains.** Strains displaying a phenotype were categorized according to their function and incorporation in protein complexes. Gene deletions confer sensitivity (red) or resistance (green) to CG-1521.

Gene ontology (GO) analysis using DAVID was used to determine which functional classes are enriched in proteins corresponding to the list of sensitive and resistant gene deletion strains [28]. Gene deletion mutants that are sensitive to CG-1521 are highly enriched in processes regulating chromatin organization and transcription (Table 1). Proteins corresponding to the gene deletion strains resistant to CG-1521 are enriched for those involved in tRNA modification (GO:0006400) and regulation of transcription, DNA-dependent (GO:0006355) and its child, negative regulation of transcription (GO:0045892). The other child, positive regulation of transcription (GO:0045893), was not significantly enriched (Table 2). Deletion of complexes associated with chromatin conformation, the HAT and HDAC complexes that modulate histone acetylation, the compass complex, which modulates histone methylation and the Swi/Snf, Swr1 and Ino80 complexes that are central to changes in chromatin conformation, all confer sensitivity to CG-1521 (Figure 2). Proteins of several other biological processes associated with transcriptional regulation, modulate the response to CG-1521, including elongation factors (THO complex, Paf1 complex, transcription elongation factor complex) and CTD kinases, as well as other modulators of transcription. Additionally, cellular machineries of translation and mRNA processing, including poly(A)-modification, mRNA degradation and splicing affect the sensitivity to CG-1521. Gene products of several other biological processes, including vacuolar acidification, vacuolar protein sorting, vesicle-mediated transport, DNA repair and cell cycle regulation (Figure 2, Additional file 2) predominantly decrease the sensitivity to CG-1521. Deletion mutants, lacking genes important for bud site selection, recovery from arrest in response to pheromone, G_1_/S and G_2_/M progression and cytokinesis are sensitive to CG-1521. Components of the Mediator and Elongator complexes are enriched in the resistant strains. Since the Elongator complex has roles in transcription elongation and wobble nucleoside modification in tRNA, it is not clear whether one or both processes are important in the response to CG-1521 (Figure 2).

**Table 1:** **Gene ontology analysis of CG-1521-sensitive strains using DAVID bioinformatics**

Gene ontology analysis: sensitive strains				
Category (GO-FAT)		p-value	Corrected p-value	Represented strains
Chromosome Organization	GO:0051276	4.5E-5	1.5E-3	49
Chromatin Organization	GO:0006325	1.2E-8	7.1E-6	41
Chromatin Modification	GO:0016568	1.4E-7	4.3E-5	35
Histone Modification	GO:0016570	6.5E-7	1.3E-4	23
Histone Exchange	GO:0043486	3.1E-4	7.0E-3	6
Transcription	GO:0006350	1.7E-6	2.1E-4	69
Regulation of Transcription	GO:0045449	7.0E-8	2.8E-5	82
Positive Regulation of Transcription	GO:0045941	6.1E-6	3.8E-4	30
Negative Regulation of Transcription	GO:0016481	1.1E-5	5.6E-4	32
Regulation of Transcription from RNA Pol II Promoter	GO:0006357	5.2E-3	6.9E-2	31
SAGA Complex	GO:0000124	6.9E-6	6.2E-4	10
Swr1 Complex	GO:0000812	1.8E-3	2.6E-2	6
Rpd3L Complex	GO:0033698	1.8E-3	2.6E-2	6

Four hundred and seven sensitive strains were identified. Reported p values have been corrected using the methods described by Benjamini and Hochberg. The GO FAT database, developed as part of DAVID, removes very broad GO terms and comprises more specific terms [28, 29].

**Table 2:** **Gene ontology analysis of CG-1521-resistant strains using DAVID bioinformatics**

Gene ontology analysis: resistant strains				
Category (GO-FAT)		p-value	Corrected p-value	Represented strains
tRNA Modification	GO:0006400	2.2E-4	1.1E-2	7
tRNA Wobble Uridine Modification	GO:0002098	6.9E-7	3.2E-4	7
Transcription	GO:0006350	1.1E-2	0.19 †	15
Regulation of Transcription, DNA-dependent	GO:0006355	2.9E-6	6.6E-4	20
Negative Regulation of Transcription	GO:0045892	6.5E-5	5.9E-3	11
Regulation of Transcription from RNA Pol II Promoter	GO:0006357	2.4E-5	2.7E-3	14
Srb Mediator Complex	GO:0016592	1.1E-5	1.7E-3	6

Eighty resistant strains were identified in the yeast gene deletion library screen. Reported p values have been corrected using the methods described by Benjamini and Hochberg. The GO FAT database, developed as part of DAVID, removes very broad GO terms and comprises more specific terms [28, 29]. †Corrected p-values not significant.

To confirm the sensitivity of the strains a secondary screen was performed in liquid culture as detailed in Methods (Additional file 3). Sixty five of seventy two tested sensitive strains were validated. These encompass gene deletion mutants that lack genes involved in transcription (CTK1-3, THO and Paf1 complex, transcription factors) and chromatin remodeling, including components of the Rpd3L (Dep1, Sin3, Pho23, Ume1, Rxt2, Sap30), Swr1 (Yaf9, Htz1, Swc5, Swc3, Arp6, Swr1, Swc2) and the Gcn5 HAT complex (Gcn5, Ada2, Ngg1, Sgf73, Spt3, Spt7, Spt8, Hfi1).

### Loss of Gcn5 HAT complexes confers sensitivity to CG-1521

**Figure 3:**
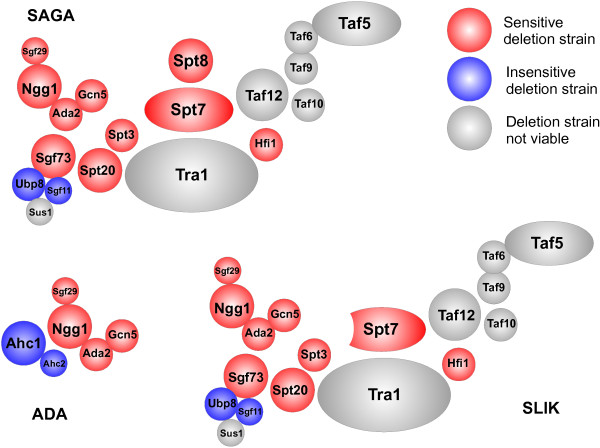
**Sensitivity of components of the Gcn5 HAT complexes.** The assembly and spatial organization of SAGA, SLIK and ADA complexes are shown, adapted from [30]. The organization of the SLIK complex was inferred from that of the SAGA complex. The SAGA complex consists of four modules, the HAT core module, the DUB module, the SPT module and the TAF module. The SLIK complex contains an additional protein Rtg2, a truncated Spt7 and is missing Spt8. The HAT module and the ADA module make up the ADA complex. Components not depicted are Chd1 and Rtg2.

Deletion mutants associated with Gcn5 HAT complexes are overrepresented (p = 6.2E-4) in the CG-1521-sensitive strains (SAGA (Spt-Ada-Gcn5-acetyltransferase) complex) (Figure 3). Gcn5 is a component of three HAT complexes in *S. cerevisiae*, the ADA, SAGA and SLIK (SAGA-like) complexes. Of the sixteen components that have corresponding deletion strains in the library, ten are sensitive (Gcn5, Ada2, Ngg1, Sgf29, Sgf73, Spt3, Spt7, Spt8, Spt20, Hfi1, red) and six are not sensitive (Ubp8, Sgf11, Chd1, Rtg2, Ahc1, Ahc2, blue). Deletion of Spt20, Spt7, Gcn5 and Ada2 results in high sensitivity to CG-1521 (scores 10–12). Gene deletion mutants *sgf29Δ*, *spt3Δ*, *spt8Δ* and *hfi1Δ* are moderately sensitive (scores 6–7) and *ngg1Δ* and *sgf73Δ* display low sensitivity (score 5 and 4). Sensitivity scores from the screen and the human homologs of the Gcn5 HAT complex components can be found in Additional file 4. The ADA, SAGA and SLIK complexes share the HAT core module, consisting of the catalytically active histone acetyltransferase Gcn5, Ada2, Ada3/Ngg1 and Sgf29. Deletion of any of these genes confers sensitivity to CG-1521 treatment. In contrast, deletion of ADA or SLIK specific components does not result in sensitivity to CG-1521, suggesting that the SAGA complex is required to reduce inhibitory effects of CG-1521 on cell growth. Deletion of the deubiquitination (DUB) module components, Ubp8 and Sgf11, does not sensitize cells to CG-1521, indicating that other functions of the SAGA complex are critical for the response to CG-1521.

**Figure 4:**
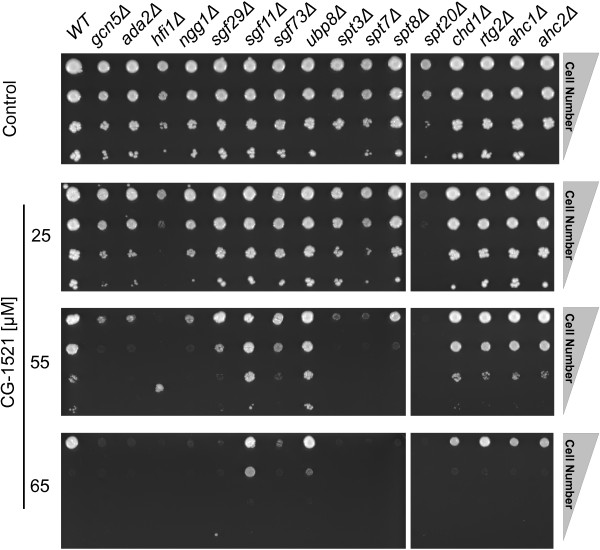
**Validation of Gcn5 HAT complex components sensitivity to CG-1521.** One μL cell suspension was spotted on CG-1521 containing agar plates, containing 25, 55 or 65 μM CG-1521, incubated for 60 h and imaged as described in Methods. The experiment was repeated three times. Gene deletion mutants *spt8Δ* and *spt20Δ* are not adjacent, however all deletion mutants are on the same agar plate. Strains lacking Gcn5, Ada2, Hfi1, Ngg1, Sgf29, Sgf73, Spt3, Spt7, Spt8 and Spt20 are sensitive to CG-1521.

The sensitivity of strains lacking components of these HAT complexes has been validated on agar plates using several starting cell densities as well as three different concentrations of CG-1521 (Figure 4). The most sensitive strains are *hfi1Δ* and *spt20Δ*, displaying sensitivity at 25 μM CG-1521. Most deletion mutants demonstrate the same sensitivity to CG-1521 as in the initial screen, however, the sensitivity of the *hfi1Δ* mutant is enhanced compared to the screen and yeast deletion mutants *ngg1Δ* and *spt3Δ* show slightly increased sensitivity.

**Figure 5:**
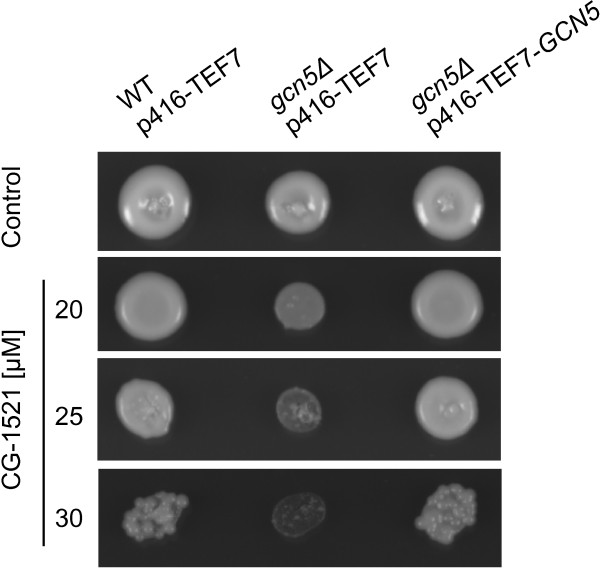
**Complementation with** ***GCN5*** **rescues from the effects of CG-1521.** BY4741 p416-TEF7, BY4741 *gcn5Δ* p416-TEF7 and BY4741 *gcn5Δ* p416-TEF7-*GCN5* were spotted on SD-URA agar plates containing 20, 25 and 30 μM CG-1521 and incubated for 60 h as described in Methods. The experiment was performed in triplicate.

To confirm that the sensitivity of the *gcn5Δ* strain is due to the loss of *GCN5*, the sensitivity of the *GCN5* complemented strain (BY4741 *gcn5Δ,* transformed with p416-TEF7-*GCN5*) was compared to the BY4741 wild-type and the *gcn5Δ* strain. Complementation with *GCN5* results in a similar level of resistance as the wild-type (Figure 5), highlighting an important role for Gcn5 in modulating the biological response to CG-1521. To assess the importance of the acetyltransferase function of Gcn5 in the attenuation of CG-1521 activity, the sensitivity of the catalytic site mutant Gcn5 *E173Q,* which has minimal residual catalytic activity [31], was measured in liquid culture. As shown in Table 3, compared to the wild-type, the *gcn5Δ* mutant is sensitive at 25 and 50 μM CG-1521. The E173Q catalytic site mutant is sensitive to CG-1521, but to a lesser extent than the *gcn5Δ* mutant (p < 0.05), suggesting that functions other than the acetyltransferase activity of Gcn5 play a role in the response to CG-1521 and may be sufficient to maintain cell growth.

**Table 3:** **Effect of CG-1521 on cell growth of wild-type,** ***gcn5Δ*** **and** ***gcn5*** **catalytic site mutant strains**

OD600 fraction of control				
CG-1521 [μM]		25	50	
BY4741	WT		0.87 ± 0.04	^†^
BY4741	*gcn5Δ*		0.26 ± 0.18	^†^
DY2396	WT	0.88 ± 0.05	0.54 ± 0.06	*
DY5925	*gcn5Δ*	0.48 ± 0.06	0.15 ± 0.04	*
DY6603	*gcn5(E173Q)*	0.71 ± 0.09	0.21 ± 0.07	*

Early-log phase BY4741 cells were treated with 25, 50 μM CG-1521 or vehicle control (DMSO) for 20 h. Cell growth was measured and the ratio of OD600 (CG-1521 cells/DMSO treated cells) was determined as described in Methods. The experiment was performed in 6 independent replicates (BY4741) or 7 independent replicates for DY2396/DY5925/DY6603 strains. The data represent the mean ± SD. (^†^,* Comparisons are considered significant if p < 0.05; within each treatment group all combinations are significantly different from each other).

### CG-1521 treatment results in G_0_/G_1_delay and deletion of *GCN5*increases susceptibility to cell death

**Figure 6:**
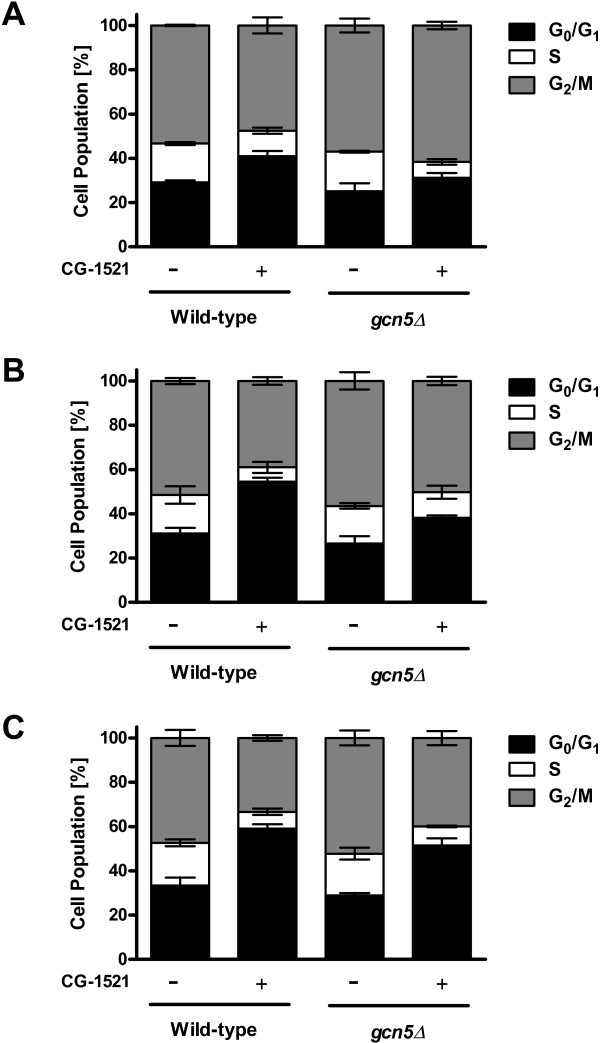
**CG-1521 induces G**_**0**_ **/G**_**1**_ **cell cycle delay in wild-type and** ***gcn5Δ*** **strains.** Exponentially growing yeast cells were treated with 50 μM CG-1521 for 1 h **(Panel A)**, 2 h **(Panel B)** or 4 h **(Panel C)**, and cell cycle profiles were measured by flow cytometry. Three independent biological replicates were performed. Data are presented as mean ± SD.

Based on the reported involvement of Gcn5 in cell cycle [32, 33], the effects of CG-1521 on cell cycle progression in wild-type and *gcn5Δ* cells were compared. The growth inhibitory effect of CG-1521 is more pronounced in the *gcn5Δ s*train than in the wild-type strain as determined on agar plates and in liquid culture. Cell cycle analysis shows that CG-1521 induces G_0_/G_1_ arrest in both strains (Figure 6). Treatment with 50 μM CG-1521 leads to a significant increase in the G_0_/G_1_ population after 1 h and 2 h for the wild-type and the *gcn5Δ s*train respectively, indicating that the growth arrest is delayed in the *gcn5Δ s*train compared to the wild-type strain. At 4 h, the G_0_/G_1_ population increases by 1.8 fold after treatment with CG-1521 in both the wild-type and the *gcn5Δ s*train. The induction of G_0_/G_1_ delay by CG-1521 was confirmed by budding index analysis (Table 4). Treatment with 50 μM CG-1521 reduces the budding index by approximately 50% in both wild-type and *gcn5Δ* strains by 2 h to 4 h*.* As a positive control for G_1_ arrest, both strains were treated with 5 μg/mL α-factor, which reduces the budding index to approximately 0.1 after 2 h in both strains.

**Table 4:** **CG-1521 induces a budding index decrease in wild-type and** ***gcn5Δ*** **strains**

Budding index analysis			
Strain	Treatment	Budding index	
		2 h	4 h
WT	Control	0.50 ± 0.05	0.48 ± 0.03
	50 μM CG-1521	0.30 ± 0.03*	0.16 ± 0.07*
	5 μg/mL α-factor	0.10 ± 0.05*	0.25 ± 0.03*
*gcn5Δ*	Control	0.44 ± 0.04	0.44 ± 0.07
	50 μM CG-1521	0.25 ± 0.04*	0.23 ± 0.07*
	5 μg/mL α-factor	0.12 ± 0.04*	0.33 ± 0.07

The budding index was determined as described in Methods. The data represent the mean ± SD of five independent biological replicates (*Comparisons are considered significant if p < 0.05 compared to control).

**Figure 7:**
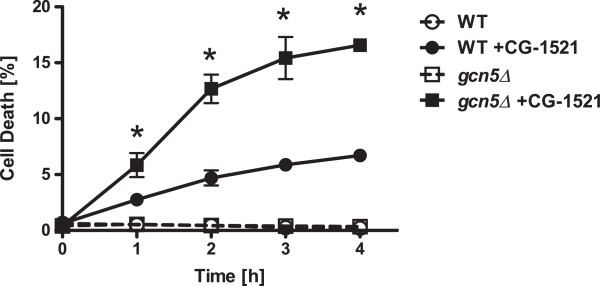
**Comparison of CG-1521-induced cell death in wild-type and** ***gcn5Δ*** **strains.** Exponentially growing yeast cells were treated with 50 μM CG-1521. Cell death was assessed by propidium iodide uptake and propidium iodide positive cells were quantitated by flow cytometry. The experiment was repeated three times. Data are presented as mean ± SD (*p < 0.05).

CG-1521 significantly induces cell death in both *gcn5Δ* and wild-type strain, as measured by propidium idodide uptake using flow cytometry. As shown in Figure 7, the *gcn5Δ* strain displays increased susceptibility to CG-1521-induced cell death compared to the wild-type strain. The difference is evident as early as 1 h after treatment with CG-1521 (p < 0.05). The *gcn5Δ* strain shows increased cell death after 1 h, whereas cell death in the wild-type strain increases significantly after 2 h. By 4 h propidium iodide uptake is detectable in 6.7% of the wild-type population, compared to 16.6% of the *gcn5Δ* population.

## Discussion

The global decrease in histone modification, particularly methylation and acetylation correlates with an aggressive phenotype and poor prognosis in a number of cancers including prostate, lung and kidney cancer [2, 3]. The ability of HDACis to induce death in a variety of cell lines is well documented, however the mechanisms by which they exert their effects are incompletely understood [34–36]. Since many biological processes are regulated by acetylation [37], we have used a yeast deletion library screen to gain insights into the cell growth inhibition mechanisms of HDAC inhibitors and to identify novel targets for combination treatments with the HDACi CG-1521. Choosing *S. cerevisiae* as a model organism decreases the complexity, however the high degree of functional homology among eukaryotes enables the identification of pathways that are important in the response to CG-1521. For example, mitotic analysis after exposure to TSA shows disruption of centromeric heterochromatin, mitotic delay and chromosome segregation defects in both fission yeast [38, 39] and mammalian cells [40, 41]. Eukaryotic cells have varied responses to HDACis, which in mammalian cells is partially dictated by the p53 status of the cell lines. For example, treatment of LNCaP prostate cancer cells, which express wild-type p53, with CG-1521 induces G_2_/M arrest and apoptosis [15]. In contrast, TSA induces G_1_/S arrest [18]. These differences in biological response have been attributed to differences in the site specific acetylation of p53, stabilized by these two drugs [19]. However, MCF-7 breast cancer cells, which express wild-type p53 and SUM190PT, which express mutant p53 both arrest in G_0_/G_1_ after treatment with either CG-1521 or TSA, suggesting that the complement of HDACs present in the cells also plays a significant role in dictating the biological outcome of treatment.

Despite the roles of histone acetyltransferases and histone deacetylases in DNA replication and DNA repair, cytoskeleton dynamics and cell cycle, these functional classes are not significantly enriched in our screen [37]. However CG-1521-sensitive strains are significantly enriched in vesicle-mediated transport, endocytosis and ubiquitin ligation (Additional file 2), which have been shown to be regulated by acetylation [37].

Previous studies have identified 63 gene deletion mutants that result in reduced H3K18 acetylation levels in *S. cerevisiae* [42]. These include genes associated with vacuolar-protein sorting, V-ATPase and SAGA complexes. Twenty-four of these 63 strains were identified as sensitive to CG-1521 in the present study. Deletion of additional genes associated with vacuolar acidification, the vacuolar proton-transporting V-type ATPase complex and vacuolar transport also renders yeast cells sensitive to CG-1521. Potentially, these CG-1521-sensitive strains are characterized by decreased histone acetylation and Gcn5 HAT activity as well. This suggests that disrupting the dynamics of acetylation and deacetylation renders cells sensitive to CG-1521.

Strains lacking components of the Gcn5 HAT complexes are very sensitive to CG-1521. Deletion of any of the four components of the histone acetyltransferase module (Sgf29, Ngg1, Ada2 or Gcn5) renders *S. cerevisiae* sensitive to CG-1521 and deletion of components of the SPT module (Spt3, Spt7, Spt8, Spt20, Hfi1) also results in increased sensitivity to CG-1521. As the Taf module components (Taf5, Taf6, Taf9, Taf10 and Taf12) and Tra1 are essential for cell survival, it is not possible to determine whether deletion of these proteins also confers sensitivity to CG-1521.

The absence of several deletion strains from the list of sensitive strains is also notable. Loss of the Ubp8 and Sgf11 components of the deubiquitination module does not sensitize the cells to CG-1521. Ubp8 and Sgf11 are part of a discrete functional module within the SAGA complex as suggested by genetic interaction and microarray analysis [43] and Ubp8 is dispensable at promoters of several SAGA-dependent genes [44]. These results suggest that the effects of CG-1521 are not modulated by the deuibiquitination activities associated with the SAGA complex. CG-1521 exhibits an increased growth inhibitory effect on the *sgf73Δ* strain compared to the wild-type. Sgf73 tethers the DUB module to the SAGA complex [45] and recruits the complex to its substrate to stimulate the formation of the pre-initiation complex [46]. It is probable that the sensitivity of the *sgf73Δ* strain to CG-1521 is due to its latter role in the formation of the pre-initiation complex.

Deletion of the SLIK specific component Rtg2, which, in association with Rtg1, Rtg3, Mks1, Lst8 and Tor1 is also responsible for mediating signaling between the mitochondrion and nucleus [47–52] does not alter the response to CG-1521. Since Rtg2 is required for SLIK integrity [53], this suggests that the SLIK complex is not necessary for eliciting a response to CG-1521. In addition, deletion of ADA (Ahc1, Ahc2) specific components does not sensitize cells to CG-1521, indicating that it is the SAGA complex, rather than ADA or SLIK complexes, that reduces the growth-inhibitory effects of CG-1521.

The SAGA complex may also act as physical adapter independent of Gcn5 and recruit TBP through Spt3 and Spt8. For example, it has been shown that H3 acetylation and HAT activity at the Gal1 promoter are not necessary for the formation of the pre-initiation complex, however pre-initiation complex assembly on the Pho84 promoter requires Gcn5 activity [44, 46]. Gene expression analysis also demonstrates that expression of distinct sets of genes is dependent on individual SAGA subunits [54]. Thus it appears that the requirement for Gcn5 activity is gene specific, suggesting that genes that require Gcn5 for their transcription are required to ameliorate the effect of CG-1521.

The data suggest that other functions of Gcn5, besides the catalytic activity, influence the response to CG-1521 since catalytic site mutation does not confer the same extent of sensitivity as *GCN5* deletion. The bromodomain of Gcn5 may recruit the HAT complex to acetylated histones. Studies in mice also indicate that there is a difference between gene deletion and catalytic site mutation. Deletion of the murine Gcn5 homolog is embryonic lethal, as the mice show increased apoptosis in mesodermal lineages. However, mouse embryos expressing a catalytically inactive protein survive significantly longer and die as a result of exencephaly [55]. These and results presented here indicate that Gcn5 has important functions that are independent of its HAT activity.

The mechanism by which CG-1521 elicits its growth-inhibitory action is probably multifactorial. As an HDAC inhibitor it differentially regulates gene expression and influences activity, stability, and assembly of protein complexes through protein acetylation. Similarly, it is likely that the SAGA complex components, including Gcn5, regulate multiple pathways in response to CG-1521, which protect the cell. Potential targets of CG-1521 that may account for the sensitivity of the *gcn5Δ* strain were analyzed through identification of negative genetic interactions with *GCN5* deletion that display insensitivity to CG-1521. Gene ontology analysis of deletion strains that are insensitive to CG-1521 and are synthetic lethal with *GCN5* deletion shows an enrichment in processes like chromatin modification, transcriptional regulation, histone acetylation, DNA repair and response to stress. Notably, deletion of components of the Rpd3 histone deacetylase complexes (Rpd3, Ash1, Pho23, Rxt2, Sap30, Sds3, Sin3 and Eaf3) results in negative genetic interactions with *GCN5* deletion, suggesting that the inhibition of Rpd3 by CG-1521 may contribute to the sensitivity of the *gcn5Δ* strain to CG-1521. The CG-1521-sensitive SAGA deletion mutants *ada2Δ*, *ngg1Δ*, *spt3Δ*, *spt7Δ*, *spt8Δ*, *spt20Δ* and *hfi1Δ* show a severe fitness defect or lethality when combined with *RPD3* deletion [43, 56–59]. However deletion mutants of components of the Rpd3L complex (*rxt2Δ*, *ume1Δ*, *pho23Δ*, *sap30Δ* and *sin3 Δ*, *dep1Δ*) are minimally or moderately sensitive to CG-1521, indicating that CG-1521 inhibits several HDACs. This correlates with the fact that none of the individual yeast HDAC deletion strains display resistance to CG-1521.

The human homologs of Gcn5, GCN5 and its paralogue PCAF (p300/CBP associated factor), the histone acetyltransferase components of the human ATAC and SAGA complexes [60], have been implicated in cancer, and these HATs are co-regulators for several proto-oncogenes [61]. The human homolog of Tra1, TRRAP, has been shown to bind c-Myc, leading to histone H4 acetylation and increased expression of Myc-dependent genes [62–64]. TRRAP interacts with the N-terminus of c-Myc [62], and truncated Myc isoforms lacking part of the N-terminal transactivation domain are transcriptionally inactive [65]. Transcriptional activation by Myc requires the GCN5 HAT activity as well as the SPT3/GCN5 interaction domain of TRRAP, suggesting that TRRAP serves as an adapter to recruit GCN5 to Myc-dependent genes [66, 67] and Myc has been reported to globally promote an active chromatin state, potentially by upregulating GCN5 expression [68]. GCN5/PCAF also directly acetylate Myc resulting in increased protein stability [69], which may provide a positive feedback loop through GCN5 upregulation and further Myc stabilization. Both TRRAP and GCN5 are required for Myc-dependent transformation [62, 66] and upregulation of Gcn5 by Myc contributes to the block of erythroid differentiation [70]. These results suggest that, GCN5 and Myc co-operate to block differentiation and promote transformation. Given the importance of GCN5 HAT complexes for Myc-dependent transcription and transformation in human cells and the synthetic lethality of *GCN5* deletion and CG-1521 treatment in yeast, it is likely that the effect of GCN5 knockdown (or inhibition of the acetyl transferase activity) combined with CG-1521 administration in Myc-driven tumors will lead to the blockade of tumor progression.

GCN5 and PCAF have been shown to regulate transcription, mediated by other transcription factors, including E2F and p53 [61, 71, 72]. Underlining the versatility of the human Gcn5 homologs, GCN5 and PCAF have been shown to interact with BRCA2 and BRCA1, respectively. These HATs have been shown to modulate BRCA-mediated DNA repair as well as their transcriptional activation function [73, 74]. GCN5 is also a potential target for oncogenic EGF signaling as it facilitates EGF mediated transcription through localized acetylation [75]. GCN5 and PCAF appear to be good targets for cancer therapy since they are associated with several proto-oncogenes and are not frequently mutated in human cancers [76–79].

Inhibitors for histone acetyltransferases are being developed and include garcinol and anacardic acid derivatives as well as synthetic inhibitors including isothiazolones, α-methylene-γ-butyrolactones, and the new pyridoisothiazolone-based inhibitors that appear to be very active inhibitors of PCAF [80–83]. The γ-butyrolactone MB-3 has been characterized as a GCN5 inhibitor *in vitro* and may be a potential treatment for acute lymphoblastic leukemia (ALL) [84, 85]. GCN5/SAGA interacts and acetylates the oncogene E2A-PBX1 resulting in protein stabilization and in this context GCN5 inhibition results in decreased levels of the E2A-PBX1 oncogene [84]. However, as is the case with HDACis, specificity will have to be precisely determined. It will be important to determine the growth-inhibitory activity of GCN5/PCAF specific inhibitors in combination with CG-1521.

## Conclusion

We have used a high throughput yeast deletion library screen to quantify strain growth after treatment with the HDACi CG-1521 and have identified 407 sensitive strains and 80 resistant strains. Biological processes including transcription and chromatin remodeling are highly represented in CG-1521-sensitive strains. In particular deletion of components of the SAGA complex, including Gcn5, confers sensitivity to CG-1521. The identification of potential pathways that modulate the response to CG-1521 in yeast will allow the evaluation of combinatorial drug targets as well as resistance markers for cancer. Based on this study we suggest that the use of HDAC inhibitors in combination with Gcn5 inhibitors may be useful for the treatment of a variety of cancers. These combination therapies may also provide novel therapeutic approaches for Myc-driven tumors.

## Methods

### Strains

The *S. cerevisiae* library (Open Biosystems, Thermo Scientific, Hudson, NH) established by the Yeast Deletion Consortium, contains 4852 gene deletion strains on the BY4741 background (Genotype: MATa his3Δ1 leu2Δ0 met15Δ0 ura3Δ0) [86]. The parental strain, transformed with pYE13G (American Type Culture Collection), conferring G418 resistance, was grown in growth media containing G418, as previously described [87].

Strains DY2396, DY5925 and DY6603 were generously provided by Dr. Stillman (University of Utah Health Science Center) [88]. Strains BY4741 p416-TEF7, BY4741 *gcn5Δ* p416-TEF7 and BY4741 *gcn5Δ* p416-TEF7-*GCN5* were generously provided by Dr. Alper (University of Texas at Austin) [89, 90] (Additional file 5).

### Yeast deletion library screen

The yeast deletion library screen was performed as previously described [91]. Briefly, 96 well plates were replicated in 150 μL YPD, containing 200 μg/mL G418. The settled cell suspension was mixed and 1 μL was spotted on agar plates containing a low (55 μM), medium (65–70 μM) and a high (70–75 μM) concentration of CG-1521 (Errant Gene Therapeutics, Chicago, IL) using the Matrix Hydra liquid handling apparatus (Thermo Scientific, Hudson, NH). Plates were imaged after 60 h incubation using the AlphaImager (Alpha Innotech Corporation, San Leandro, CA). The wild-type strain and the positive control strain (*spt3Δ*) were also spotted on each plate. The screen was performed twice (Table 5). Sensitivity and resistance was scored relative to the non-treated control and wild-type growth. Depending on the degree of sensitivity, strains were attributed a score from 1 to 3, while resistant strains were scored on a scale of -1 to -2. The sum of these scores across CG-1521 concentrations and biological replicates yields the final score for the respective strain. Strains with a score of ≥ 3 were regarded as sensitive, while strains with a score of ≤ -2 were regarded as resistant. Gene Ontology Analysis was performed using DAVID Bio-informatics Resources (Database for Annotation, Visualization and Integrated Discovery), reported p values have been corrected for False Discovery Rate using the methods described by Benjamini and Hochberg [28]. The screening methodology, which scores mutants as sensitive or resistant compared to the non-treated and the wild-type strain, cannot completely account for the differences in growth rates and morphologies of the deletion strain. While many of the slower growing deletion strains are not sensitive to CG-1521, the possibility that some of the sensitive strains are hypersensitive to CG-1521, due in part to their compromised growth, can not be excluded. For this reason, the sensitivity of the SAGA complex deletion strains was verified in liquid culture and agar spot assays.

**Table 5:** **Study design**

Experimental design	
Plates	57
Replicates	2
Conditions	0 (Control)
CG-1521 [μM]	55
	67.5
	72.5
Strains	4852
Total number of plates	456
Total number of data points	39,729

The yeast gene deletion library encompasses 57 plates (4852 strains). The complete screen was performed twice with three different concentrations of CG-1521 (55 μM, 67.5 μM and 72.5 μM).

### Validation using an agar spot assay

Sensitivity of gene deletion strains specific to the Gcn5 HAT complex was verified as described above. Strains were spotted on agar plates containing 25 μM, 55 μM or 65 μM CG-1521. Different cell concentrations were spotted using 1:20 serial dilution. Experiments using BY4741 p416-TEF7 strains were performed in synthetic dropout media lacking uracil, and sensitivity was determined using 20, 25 and 30 μM CG-1521.

### Validation in liquid culture

The sensitivity of strains harboring gene deletions corresponding to proteins with functions in chromatin remodeling and transcriptional regulation was verified in liquid culture. 195 μL YPD containing 25 μM, 50 μM or 65 μM CG-1521 were inoculated with 5 μL cell suspension. After 20 h incubation, the cell suspension was diluted 1:2 and the OD_600_ was measured. To normalize for differences in growth between strains the strain growth relative to wild-type cells was calculated and the ratio of treated versus untreated control was determined and expressed as Net Treated Growth Value (NTGV). Strains with NTGVs ≤ 0.7 are regarded as sensitive. Strains with NTGVs ≥ 1.2 are regarded as resistant.

Exponentially growing yeast cultures (BY4741 wild-type, BY4741 *gcn5Δ*, DY2396, DY5925, DY6603) were treated with 25 or 50 μM CG-1521 or vehicle control (DMSO) for 20 h, the OD_600_ was determined and the ratio (treated/untreated) was calculated.

### Cell cycle analysis

Yeast cells, growing in log phase, were treated with 50 μM CG-1521 or vehicle control (DMSO) for 1 to 4 h. After fixation in 100% ethanol for 12–16 h, cells were washed and 10^7^ cells were resuspended in 1 mL 50 mM sodium citrate pH 7.0 containing 100 μg/mL RNase A (Sigma, St. Louis, MO), incubated overnight at 55°C and treated with proteinase K (0.1 mg/mL, Amresco, Solon, OH) for 5 h at 55°C. The cells were stained with 5 μL 1 mg/mL propidium iodide for 30 min and the DNA content was analyzed by flow cytometry using a BD LSR2 flow cytometer (BD Biosciences, San Jose, CA). The data were analyzed using FloJo™ software (Tree Star, Inc., Ashland, OR).

### Budding index analysis

Flow cytometry results were confirmed by Budding Index Analysis. Exponentially growing yeast cultures (10^7^ cells/mL) were treated with 50 μM CG-1521, DMSO or 5 μg/mL α-factor (Sigma). The budding index was calculated by counting the number of unbudded and budded cells in approximately 100 yeast cells for each treatment condition.

### Cell death analysis

*S. cerevisiae* were cultured as described above for cell cycle analysis. Aliquots were removed from the culture at 0, 2 and 4 h. The cell suspension was pelleted and resuspended in phosphate buffered saline and incubated with 1 μL propidium iodide (PI) for 10 min in the dark. PI uptake was quantitated by flow cytometry analysis using a BD LSR2 flow cytometer (BD Biosciences). The data were analyzed using FloJo™ software.

### Statistical analysis

For all experiments, three or more independent biological replicates were performed. The results are presented as mean ± SD. Results are regarded significant if p < 0.05 as established by ANOVA and Tukey-Kramer post-test.

## Authors’ information

ACG is a graduate student in the Department of Biomedical Sciences, School of Public Health, University at Albany and a member of the Cancer Research Center. TB is a member of the Nanobioscience Constellation in the SUNY College of Nanoscale Science and Engineering. MT is a member of the Department of Biomedical Sciences, School of Public Health, University at Albany and Director of the Cancer Research Center.

## Electronic supplementary material

Additional file 1: **Sensitive and Resistant Strains.** Strains are sorted according to their sensitivity or resistance to CG-1521 compared to the wild-type strain. (XLS 90 KB)

Additional file 2: **Additional GO-Terms - Sensitive Strains.** Gene ontology analysis using DAVID Bioinformatics of CG-1521 sensitive strains indicates an enrichment in ontologies, including vesicle-mediated transport and vacuolar acidification, purine nucleotide biosynthetic process, ubiquitin ligase complexes and cytoplasmic mRNA processing body in addition to chromatin remodeling and transcription. †Corrected p-values are not significant. (DOCX 14 KB)

Additional file 3: **Validation of Sensitivity.** Validation of the sensitivity of CG-1521-sensitive strains in liquid culture. The net treated growth value was calculated to normalize for strain growth differences relative to the control. The data represent 3 independent experiments. (XLSX 15 KB)

Additional file 4: **Components of the Gcn5 HAT complexes and their human orthologs.** The SAGA complex consists of four modules, the HAT core module (green), the DUB module (purple), the SPT module (orange) and the TAF module (blue). The SLIK complex contains an additional protein Rtg2, a truncated Spt7 and is missing Spt8. The HAT module and the ADA module (red) make up the ADA complex. GO-Analysis shows an enrichment of sensitive strains in the Gcn5 HAT complexes. Sensitive components are represented in red, insensitive deletion strains in blue. Proteins depicted in black are not present in the yeast deletion library. (DOCX 16 KB)

Additional file 5: **Yeast Strains.** Yeast strains were generously provided by Dr. Stillman (*) 88 and Dr. Alper (^#^) [89, 90] respectively. (DOCX 13 KB)

## Abbreviations

HDAC: Histone deacetylase
HDACi: Histone deacetylase inhibitor
HAT: Histone acetyltransferase
*S. cerevisiae*: *Saccharomyces cerevisiae*
TSA: Trichostatin A
SAHA: Suberoylanilide hydroxamic acid
GO: Gene ontology
DAVID: Database for annotation, visualization and integrated discovery
SAGA complex: Spt-Ada-Gcn5-acetyltransferase complex
SLIK complex: SAGA-like complex
DUB module: Deubiquitination module
ALL: Acute lymphoblastic leukemia.
