# Supplementary material for: High throughput screening identifies modulators of histone deacetylase inhibitors
Source: BMC Genomics. 2014 Jun 26;15(1):528. doi: 10.1186/1471-2164-15-528 (PMC4089024; doi:10.1186/1471-2164-15-528)
Supplement: Supplementary file 2 — Additional file 2: Additional GO-Terms - Sensitive Strains. Gene ontology analysis using DAVID Bioinformatics of CG-1521 sensitive strains indicates an enrichment in ontologies, including vesicle-mediated transport and vacuolar acidification, purine nucleotide biosynthetic process, ubiquitin ligase complexes and cytoplasmic mRNA processing body in addition to chromatin remodeling and transcription. †Corrected p-values are not significant. (DOCX 14 KB) [file 12864_2014_6208_MOESM2_ESM.docx]

| Gene Ontology Analysis: Sensitive Strains | | |  |  |
| --- | --- | --- | --- | --- |
| Category (GO-FAT) | | p-value | Corrected p-value | Represented Strains |
| Golgi Apparatus | GO:0005794 | 2.5E-7 | 4.4E-5 | 40 |
| Endoplasmic Reticulum | GO:0005783 | 5.5E-5 | 2.2E-3 | 53 |
| Vesicle-Mediated Transport | GO:0016192 | 2.1E-5 | 8.0E-4 | 49 |
| Vacuolar Acidification | GO:0007035 | 3.1E-6 | 2.9E-4 | 11 |
| Vacuolar Proton-Transporting  V-type ATPase Complex | GO:0016471 | 4.5E-5 | 2.0E-3 | 8 |
| Vacuolar Transport | GO:0007034 | 1.7E-3 | 2.9E-2 | 19 |
| Ion Homeostasis | GO:0050801 | 1.5E-6 | 2.0E-4 | 30 |
| Endocytosis | GO:0006897 | 2.2E-3 | 3.6E-2 | 16 |
| Purine Nucleotide Biosynthetic Process | GO:0006163 | 1.0E-6 | 1.5E-4 | 20 |
| Cytoplasmic Ubiquitin Ligase Complex | GO:0000153 | 1.7E-5 | 1.0E-3 | 7 |
| Ubiquitin-Dependent Protein Catabolic Process | GO:0006511 | 2.2E-3 | 3.6E-2 | 25 |
| Cytoplasmic mRNA Processing Body | GO:0000932 | 4.7E-3 | 4.5E-2 | 7 |
| Cellular Response to Unfolded Protein | GO:0034620 | 5.0E-3 | 6.8E-2† | 6 |
| Re-Entry into Mitotic Cell Cycle | GO:0000320 | 3.5E-3 | 5.1E-2† | 5 |
| Regulation of Translation | GO:0006417 | 4.6E-2 | 3.2E-1† | 20 |
