# Supplementary material for: High throughput screening identifies modulators of histone deacetylase inhibitors
Source: BMC Genomics. 2014 Jun 26;15(1):528. doi: 10.1186/1471-2164-15-528 (PMC4089024; doi:10.1186/1471-2164-15-528)
Supplement: Supplementary file 4 — Additional file 4: Components of the Gcn5 HAT complexes and their human orthologs. The SAGA complex consists of four modules, the HAT core module (green), the DUB module (purple), the SPT module (orange) and the TAF module (blue). The SLIK complex contains an additional protein Rtg2, a truncated Spt7 and is missing Spt8. The HAT module and the ADA module (red) make up the ADA complex. GO-Analysis shows an enrichment of sensitive strains in the Gcn5 HAT complexes. Sensitive components are represented in red, insensitive deletion strains in blue. Proteins depicted in black are not present in the yeast deletion library. (DOCX 16 KB) [file 12864_2014_6208_MOESM4_ESM.docx]

| **ADA Complex** | **SAGA Complex** | **SLIK Complex** | **Sensitivity Score** | **Human Homolog** |
| --- | --- | --- | --- | --- |
| Gcn5 | Gcn5 | Gcn5 | 10 | GCN5, PCAF |
| Ada2 | Ada2 | Ada2 | 10 | TADA2B |
| Ngg1 | Ngg1 | Ngg1 | 5 | TADA3 |
| Sgf29 | Sgf29 | Sgf29 | 7 | CCDC101 |
|  |  |  |  |  |
|  | Sgf11 | Sgf11 |  | ATXN7L3 |
|  | Sgf73 | Sgf73 | 4 | ATXN7 |
|  | Ubp8 | Ubp8 |  | USP22 |
|  | Sus1 | Sus1 |  | ENY2 |
|  |  |  |  |  |
|  | Spt3 | Spt3 | 6 | SUPT3H |
|  | Spt7 | Spt7 (truncated) | 11 | SUPT7L |
|  | Spt8 |  | 6 |  |
|  | Spt20 | Spt20 | 12 | SUPT20H |
|  | Hfi1 | Hfi1 | 6 | TADA1 |
|  | Tra1 | Tra1 |  | TRRAP |
|  |  |  |  |  |
|  | Taf5 | Taf5 |  | TAF5L |
|  | Taf6 | Taf6 |  | TAF6L |
|  | Taf9 | Taf9 |  | TAF9 |
|  | Taf10 | Taf10 |  | TAF10 |
|  | Taf12 | Taf12 |  | TAF12 |
|  |  |  |  |  |
|  | Chd1 | Chd1 |  | CHD1, CHD2 |
|  |  | Rtg2 |  |  |
|  |  |  |  |  |
| Ahc1 |  |  |  |  |
| Ahc2 |  |  |  |  |
