# Supplementary material for: High throughput screening identifies modulators of histone deacetylase inhibitors
Source: BMC Genomics. 2014 Jun 26;15(1):528. doi: 10.1186/1471-2164-15-528 (PMC4089024; doi:10.1186/1471-2164-15-528)
Supplement: Supplementary file 5 — Additional file 5: Yeast Strains. Yeast strains were generously provided by Dr. Stillman (*) 88 and Dr. Alper (#) [89, 90] respectively. (DOCX 13 KB) [file 12864_2014_6208_MOESM5_ESM.docx]

| Strain | | Genotype | Isogenic Background |
| --- | --- | --- | --- |
| *DY2396 | MATa *ade2 can1 his3 leu2 lys2 trp1 ura3* | | W303 |
| *DY5925 | MATa *gcn5::HIS3 ade2 can1 his3 leu2 trp1 ura3* | | W303 |
| *DY6603 | MATa *gcn5::HIS3 URA3::GCN5(E173Q) ade2 can1 his3 leu2 trp1* | | W303 |
| ^#^ BY4741  p416-TEF7 | MATa *his3Δ1 leu2Δ0 met15Δ0 ura3Δ0* | | BY4741 |
| ^#^ BY4741 *gcn5Δ*  p416-TEF7 | MATa *gcn5::KAN his3Δ1 leu2Δ0 met15Δ0 ura3Δ0* | | BY4741 |
| ^#^ BY4741 *gcn5Δ*  p416-TEF7 GCN5 | MATa *gcn5::KAN his3Δ1 leu2Δ0 met15Δ0 ura3Δ0* | | BY4741 |
